# The effect of galantamine on brain atrophy rate in subjects with mild cognitive impairment is modified by apolipoprotein E genotype: post-hoc analysis of data from a randomized controlled trial

**DOI:** 10.1186/alzrt275

**Published:** 2014-07-21

**Authors:** Niels D Prins, Wiesje A van der Flier, Dirk L Knol, Nick C Fox, H Robert Brashear, Jeffrey S Nye, Frederik Barkhof, Philip Scheltens

**Affiliations:** 1 Alzheimer Centre, Department of Neurology, VU University Medical Centre, PO Box 7057, 1007 MB Amsterdam, the Netherlands; 2 Alzheimer Research Center, Gebouw Cronenburg, Cronenburg 75, 1081 GM Amsterdam, the Netherlands; 3 Department of Epidemiology & Biostatistics, VU University Medical Centre, PO Box 7057, 1007 MB Amsterdam, the Netherlands; 4 Dementia Research Centre (N.C.F.), Department of Neurodegenerative Disease, Institute of Neurology, University College London, 8-11 Queen Square, London WC1N 3BG, UK; 5 Janssen Alzheimer Immunotherapy Research and Development, 700 Gateway Blvd, South San Francisco, CA 94080, USA; 6 Janssen Research and Development, LLC Johnson and Johnson Innovation, One Cambridge Center, 7th Floor, Cambridge, MA 02142, USA; 7 Department of Radiology, Image Analysis Centre, VU University Medical Centre, PO Box 7057, 1007 MB Amsterdam, the Netherlands

## Abstract

**Introduction:**

The aim of this investigation was to assess the effect of galantamine, an acetylcholinesterase inhibitor and allosteric modulator of nicotinic receptors, on brain atrophy in individuals with mild cognitive impairment (MCI), and to assess effect modification by apolipoprotein E (APOE) genotype.

**Methods:**

We used data from the Galantamine-International-11 (Gal-Int-11) trial, a 24-month, randomized, double blind, placebo-controlled, flexible-dose (16 to 24 mg daily) study in patients with MCI. Brain magnetic resonance imaging (MRI), including a 3-dimensional T1-weighted gradient echo volumetric sequence, was performed at screening and at 24 months. We recorded whole brain and hippocampal volumes, and calculated annual atrophy rates. Linear regression analysis was used to calculate adjusted mean differences in the rate of whole brain and hippocampal atrophy, between MCI patients treated with galantamine and with placebo. Additionally, we performed stratified analyses according to APOE genotype.

**Results:**

Data from 364 MCI patients with 24-month MRI data (galantamine, n = 176; placebo, n = 188) were included in the volumetric analysis. Subjects treated with galantamine demonstrated a lower rate of whole brain atrophy compared to those treated with placebo (adjusted mean difference 0.18% per year (95% confidence interval (CI) 0.04; 0.30)). Stratified analyses according to APOE genotype, showed that this effect was confined to patients who carried an APOE ϵ4 allele (adjusted mean difference 0.28% per year (95% CI 0.07; 0.50)). Rates of hippocampal atrophy did not differ significantly between study groups.

**Conclusions:**

Patients with MCI who were treated with galantamine demonstrated a lower rate of whole brain atrophy, but not of hippocampal atrophy, over a 24-month treatment period, compared to those treated with placebo. This protective effect of galantamine on whole brain atrophy rate in MCI was only present in APOE ϵ4 carriers.

## Introduction

Mild cognitive impairment (MCI) is a heterogeneous syndrome characterized by a level of cognitive function (typically memory) that is worse than expected based on age and educational level, but which does not meet clinical criteria for dementia [1]. Patients with MCI have an increased risk for the development of Alzheimer’s disease (AD), with up to 15% of these patients progressing to dementia per year, compared with up to 2% of the normal older population [2,3]. Magnetic resonance imaging (MRI) has contributed to our understanding of the brain changes associated with MCI and AD. Brain atrophy is a pathologic change characteristic of AD, with results of cross-sectional and longitudinal brain imaging studies demonstrating progressive reduction in whole brain volumes and volumes of the amygdala, hippocampus, and parahippocampal gyrus [4-6]. At a group level, the degree and rate of medial temporal lobe and brain atrophy in individuals with MCI is greater than that in normal controls, and less than that in patients with AD [4]. In MCI subjects a lower brain or hippocampal volume or a higher rate of brain or hippocampal atrophy is predictive of progression of MCI to AD [7-9].

Galantamine is an acetylcholinesterase inhibitor and allosteric modulator of nicotinic receptors [10-12] that has consistently demonstrated benefits on cognition, global functioning, and the ability to perform activities of daily living in patients with mild to moderate AD [13-18]. Some preclinical studies suggest that galantamine has neuroprotective effects, the mechanism(s) of which appears to be independent of cholinesterase inhibition and possibly related to alpha-7 nicotinic receptors and the phosphatidylinositide 3-kinase–Akt pathway [19]. Since previous studies showed that MCI patients who carry an apolipoprotein E (APOE) ϵ4 allele are at a higher risk of progressing to AD and show higher rates of whole brain and hippocampal atrophy, any assessment of the effect of galantamine on atrophy in MCI should take into account the APOE genotype [20,21].

Data from a large clinical trial, conducted from 2001 to 2003, of galantamine effects in MCI were available for analysis [22]. In this trial, galantamine did not meet the primary efficacy endpoint; that is, did not reduce the percentage of subjects who converted from MCI to dementia (Clinical Dementia Rating score ≥1.0) over 2 years. However, the data from this trial are a robust source of longitudinal data on treatment effects of galantamine in patients with MCI. The objective of the current analysis was to assess the effect of galantamine (compared with placebo) on the rate of total brain and hippocampal atrophy, using serial MRI in individuals with MCI, and to assess whether this effect was modified by APOE genotype.

## Methods

### Study design and subjects

For the current prospective follow-up study, we used data from MCI patients who participated in the Galantamine-International-11 (Gal-Int-11) trial (NCT00236431). Gal-Int-11 was a 24-month, randomized, double-blind, placebo-controlled clinical trial studying the effect of galantamine on cognitive decline in subjects with MCI (Johnson & Johnson Pharmaceutical Research & Development, LLC, Titusville, NJ, USA) [22]. Patients eligible for participation in the Gal-Int-11 trial were ≥50 years of age with a history of cognitive decline but with insufficient impairment in activities of daily living to meet diagnostic criteria for dementia. Major inclusion criteria included a global Clinical Dementia Rating score of 0.5, with a memory score ≥0.5 and a delayed recall score ≤10 on the New York University paragraph recall test [23,24].

In total, 995 eligible patients were randomized (1:1 ratio) to receive galantamine or placebo for 24 months. Galantamine was administered at a dose of 4 mg twice daily for 1 month, then 8 mg twice daily for 1 month. If well tolerated, the dose could be titrated to 12 mg twice daily, but could be lowered back to 8 mg twice daily after 1 month if necessary. The dose selected at month 3 (8 mg or 12 mg twice daily) was fixed for the remainder of the 24-month study. Subjects in either study group who progressed from MCI to dementia (defined as a Clinical Dementia Rating score ≥1.0) were terminated from the double-blind phase of the study and were eligible for open-label treatment with galantamine 8 to 12 mg twice daily throughout the 24 months. The study was approved by Independent Ethics Committees or Institutional Review Boards, listed in the Appendix, and conducted in accordance with the Declaration of Helsinki and its subsequent revisions.

### Magnetic resonance imaging procedures

The MRI analysis set included 364 subjects (galantamine, *n* = 176; placebo, *n* = 188) who had taken the double-blind study drug for at least 600 days and had a suitable MRI at baseline and at 24 months. Also included were data from subjects who withdrew or started the open-label study drug but had a 24-month MRI taken at or after day 600 within 30 days of the last dose of double-blind study drug. Of the 364 subjects, 269 had a reliable whole brain atrophy change measurement, and 321 had a reliable hippocampal atrophy change measurement.

Patients underwent brain MRI at screening/baseline and at month 24 on 1.5 T scanners (GE, Philips, or Siemens). A three-dimensional T1-weighted gradient echo volumetric sequence (coronal, 1.5 mm slice thickness, 1 mm in-plane resolution) and a two-dimensional fast fluid-attenuated inversion recovery sequence (axial, 5 mm contiguous slice, 1 mm in-plane resolution) were obtained at both time points. At screening, a T2-weighted sequence also was obtained. After quality assessment, the baseline and month 24 image sets were sent on electronic media to two image analysis centers that acted as blinded central readers.

The Dementia Research Group at the National Hospital for Neurology in Queen Square, London, UK was responsible for reading the whole brain volume and for calculating the rate of brain atrophy using the brain boundary shift integral (BBSI) [25]. The rate of brain atrophy (percent change per year) was calculated as the BBSI for each individual subject, divided by the time interval in days, normalized to 1 year (365.25 days). The BBSI uses voxel intensities to determine the total volume through which an entire boundary of a structure shifts in registered serially acquired scans.

The same dataset was sent to the Image Analysis Centre at the VU University Medical Centre, Amsterdam, the Netherlands for blinded central reading of the hippocampal volume.

Hippocampal volume measurements were performed using high-resolution T1-weighted images, reformatted in a plane perpendicular to the long axis of the hippocampus. Follow-up images were registered to the baseline scan. Using 1.5 mm thick reformatted slices, approximately 20 to 25 slices were used to delineate the hippocampus. All measurements were obtained on the left and right sides separately, and were corrected for the size of the intracranial cavity (to account for premorbid differences in brain size). Hippocampal measurements were performed according to the definitions of Jack [26], using the in-house developed software package Show_Images 3.7.0, as described previously [27]. The rate of hippocampal atrophy (percent change per year) was calculated as the linear change of the hippocampal volume, divided by the time interval in days, normalized to 1 year (365.25 days).

Raters were blinded to treatment allocation, but not to the order of scanning. All subjects provided written informed consent before enrollment. The studies were approved by an Independent Ethics Committee or Institutional Review Board and were conducted in accordance with the Declaration of Helsinki and its subsequent revisions.

### Apolipoprotein E genotyping

APOE genotyping was performed as an exploratory measure in subjects who consented to participate; this participation was not mandatory for enrollees. Of the 364 subjects in the MRI analysis set, information on the APOE genotype was available for 274 subjects (75%).

### Data analysis

Analyses were performed using SPSS (version 20; SPSS, Chicago, IL, USA). The MRI analysis set comprised 364 subjects. Of those, 176 received galantamine and 188 received placebo. Information on the APOE genotype was available for 274 patients (APOE noncarriers, *n* = 145; APOE carriers, *n* = 129). We compared baseline characteristics between patients treated with galantamine (*n* = 176) and placebo (*n* = 188) and between APOE carriers and APOE noncarriers using Student’s *t* test for continuous variables and chi-square tests for nominal variables.

We assessed the associations of age, sex, vascular risk factors, baseline Alzheimer’s Disease Assessment Scale – cognitive subscale (ADAS-Cog)/MCI score, and APOE genotype with whole brain atrophy rate and hippocampal atrophy rate using linear regression analysis, adjusting for age and sex.

Of the 364 subjects in the MRI analysis set, 269 had a reliable whole brain atrophy change measurement and 321 a reliable hippocampal atrophy change measurement. We calculated age and sex-adjusted mean differences (95% confidence interval (CI)) in total brain atrophy rate and hippocampal atrophy rate between patients who received galantamine and those who received placebo, using linear regression analysis. To control for potential confounding factors, we added vascular risk factors and baseline ADAS-Cog/MCI score to the model. We repeated the analyses after stratification according to APOE genotype. Furthermore, we calculated age and sex-adjusted mean differences (95% CI) in total brain atrophy rate and hippocampal atrophy rate between APOE ϵ4 carriers and APOE ϵ4 noncarriers.

## Results

Table 1 summarizes the baseline demographic and cognitive characteristics of the 364 subjects in the MRI analysis set, according to treatment group and for the total number of subjects. The percentage of women in the placebo group was higher compared with that in the galantamine group, but there were no other differences in baseline characteristics between the placebo and galantamine-treated groups (Table 1). Of the 364 subjects in the MRI analysis set, 10 (2.7%) converted to dementia. Table 2 summarizes the same baseline characteristics, but only for the 274 subjects in the MRI analysis set who had available information on APOE genotype, separate for APOE ϵ4 carriers and APOE ϵ4 noncarriers, and for the total number. MCI patients who were APOE ϵ4 noncarriers more often had hypertension and had lower ADAS-Cog/MCI scores than MCI patients who were APOE ϵ4 carriers (Table 2).

**Table 1:** Baseline characteristics for the total number of study subjects and according to treatment group

	**Total (*n*** **= 364)**	**Placebo (*n*** **= 188)**	**Galantamine (*n*** **= 176)**	***P *** **value**^**a**^
Age	68 (8.8)	69 (8.6)	68 (9.0)	0.13
Sex, female	49	54	43	0.04
Hypertension	44	45	43	0.62
Diabetes	7	7	7	0.98
Elevated cholesterol	34	35	32	0.59
Smoking	9	10	7	0.46
ADAS-Cog/MCI	15 (7.0)	14 (6.9)	15 (7.2)	0.56
Whole brain (ml)	1,089 (124)	1,081 (124)	1,097 (123)	0.22
Hippocampal volume (ml)	5.85 (0.9)	5.78 (0.9)	5.93 (0.9)	0.11

Data presented as means (standard deviation) or percentages. ADAS-Cog, Alzheimer’s Disease Assessment Scale – cognitive subscale; MCI, mild cognitive impairment. ^a^*P* values for differences in means or percentages between patients treated with placebo and those treated with galantamine.

**Table 2:** Baseline characteristics for subjects who had available information on apolipoprotein E genotype

	**Total (*n*** **= 274)**	**APOE ϵ4 carriers (*n*** **= 129)**	**APOE ϵ4 noncarriers (*n*** **= 145)**	***P *** **value**^**a**^
Age	68 (8.9)	69 (8.8)	68 (8.9)	0.76
Sex, female	48	53	44	0.16
Hypertension	44	36	50	0.02
Diabetes	8	8	9	0.72
Cholesterol	34	30	38	0.18
Smoking	8	6	10	0.29
ADAS-Cog/MCI	14 (7.0)	16 (7.4)	13 (6.2)	0.00
Whole brain (ml)	1,088 (122)	1,083 (110)	1,092 (132)	0.54
Hippocampal volume (ml)	5.91 (0.85)	5.83 (0.90)	5.98 (0.83)	0.17

Data presented as means (standard deviation) or percentages. ADAS-Cog, Alzheimer’s Disease Assessment Scale – cognitive subscale; APOE, apolipoprotein E; MCI, mild cognitive impairment. ^a^*P* values for differences in means or percentages between patients treated with placebo and those treated with galantamine.

The mean whole brain atrophy rate was 0.52% per year and the mean hippocampal atrophy rate was 1.74% per year. Older age was associated with higher whole brain atrophy rate (β per year increase, 0.02% (95% CI, 0.01 to 0.03%)) and higher hippocampal atrophy rate (β per year increase, 0.05% (95% CI, 0.03 to 0.07%)). Presence of diabetes was associated with higher whole brain atrophy rate (β, 0.25% (95% CI, 0.05 to 0.49%)), but not with hippocampal atrophy rate. Higher ADAS-Cog/MCI score was associated with higher whole brain atrophy rate (β per point increase, 0.01% (95% CI, 0.00 to 0.02)) and higher hippocampal atrophy rate (β, 0.07% (95% CI, 0.04 to 0.10%)). APOE ϵ4 carriers had higher whole brain atrophy rates (adjusted mean difference, 0.16% (95% CI, 0.02 to 0.30%)) and hippocampal atrophy rates (adjusted mean difference, 0.68% (95% CI, 0.28 to 1.07%)) compared with APOE ϵ4 noncarriers.

Table 3 presents the difference in whole brain atrophy rate and hippocampal atrophy rate between subjects treated with placebo and those treated with galantamine. Subjects treated with galantamine demonstrated a lower rate of whole brain atrophy, but not of hippocampal atrophy, compared with those treated with placebo (Table 3). Table 4 presents the difference in whole brain atrophy rate stratified according to APOE genotype. This stratified analysis showed that the association between galantamine treatment and lower whole brain atrophy rate was confined to carriers of an APOE ϵ4 allele (Table 4). MCI patients who carried an APOE ϵ4 allele and were treated with galantamine showed 36% less whole brain atrophy compared with those who were treated with placebo. Additional adjustments for vascular risk factors and baseline ADAS-Cog/MCI score did not change the results (data not shown). There were no statistically significant between-group differences in the rate of hippocampal atrophy after stratification for APOE genotype (data not shown).

**Table 3:** Whole brain atrophy rates for subjects treated with placebo and with galantamine

	**Placebo**	**Galantamine**	**Adjusted mean difference**	**95% CI**
Whole brain atrophy rate	0.63	0.46	0.17	0.04 to 0.30
Hippocampal atrophy rate	1.70	1.94	−0.24	−0.62 to 0.12

Data presented as percentage change per year. The numbers of subjects per cell are as follows: whole brain atrophy rate, total *n* = 242 (placebo, *n* = 130; galantamine, *n* = 112); hippocampal atrophy rate, total *n* = 302 (placebo, *n* = 160; galantamine, *n* = 142). CI, confidence interval.

**Table 4:** Whole brain atrophy rates for subjects treated with placebo or galantamine, stratified according to apolipoprotein E genotype

	**Placebo**	**Galantamine**	**Adjusted mean difference**	**95% CI**
APOE ϵ4 noncarriers	0.48	0.41	0.07	−0.10; 0.25
APOE ϵ4 carriers	0.77	0.49	0.28	0.07; 0.50

Data presented as percentage change per year. The numbers of subjects per cell were as follows: APOE ϵ4 noncarriers, total *n* = 109 (placebo, *n* = 54; galantamine, *n* = 55), APOE ϵ4 carriers, total *n* = 97 (placebo, *n* = 54; galantamine, *n* = 43). APOE, apolipoprotein E; CI, confidence interval.

## Discussion

In a subset of MCI patients who participated in the Gal-Int-11 trial and underwent MRI at baseline and at 24 months, we found that MCI patients who received galantamine showed lower whole brain atrophy rates compared with those treated with placebo. In subsequent stratified analyses, we found that this difference was only present in patients who carried an APOE ϵ4 allele. We found no treatment effects on the rate of hippocampal atrophy. When interpreting these results, one should take into account that this MRI analysis set did not include MCI patients who converted to dementia before 30 days of their 24-month MRI, and that this selective dropout has contributed to a benign study sample. This explains why the mean whole brain and hippocampal atrophy rate were lower compared with other studies in MCI [8,9]. The study included patients with amnestic MCI, although it is not possible to rule out the possibility that some patients had multidomain MCI.

MRI has emerged as a useful tool for the study of MCI and AD [28]. To date, no study has evaluated the effect of galantamine on the rate of brain volume in patients with MCI. The Gal-Int-11 trial used established imaging techniques [29] and structural boundary definitions [26,30] for the study of brain morphology. The use of BBSI to determine the rate of brain atrophy minimized the impact of any inaccuracies in segmentation and operator/reader bias [25].

It is unclear why the effects of galantamine were observed on brain volume but not on hippocampal volume. Variability in hippocampal volume was possibly magnified because of the small volume of the hippocampus, making the detection of any treatment effect difficult. The absence of a significant effect on hippocampal volume might also be attributable to the lack of matching of the study groups with regard to baseline hippocampal size or a lack of power to detect a treatment effect for hippocampal atrophy. Alternatively, the effect of galantamine on brain volume may be explained by a ceiling effect.

We found that galantamine only lowered the rate of brain atrophy in MCI patients who were APOE ϵ4 carriers. Compared with noncarriers, these MCI patients are more likely to have underlying AD pathology; that is, they are more likely to classify as prodromal AD according to the Albert criteria, since APOE ϵ4 positivity in MCI has been found to be associated with amyloid positron emission tomography positivity, and with the AD biomarker profile in cerebrospinal fluid [31-33]. Our results suggest that a potential beneficial effect of galantamine on brain atrophy may be restricted to MCI patients with underlying AD pathology.

Acetylcholinesterase inhibitors have previously been shown to exhibit a neuroprotective effect via the nicotinic acetylcholine receptor-mediated cascade [34,35]. Acetylcholinesterase inhibitors that are not positive allosteric modulators of nicotinic receptors, however, have not shown significant effects on brain or hippocampal volume loss in placebo-controlled studies of 2 to 4 years’ duration in MCI patients [36,37]. In addition, it has been reported that acetylcholinesterase inhibitors inhibit the progress of brain atrophy in AD, indicating the attenuation of neuronal death in the brain of the patients, although in this study the hippocampal atrophy rate in AD patients treated with donepezil was compared with that of historic controls, and not with patients randomized to placebo in a double-blind clinical trial [21].

The reduced rate of whole brain atrophy observed in this trial provides clinical evidence of neuroprotection with galantamine treatment. Further research is needed to elucidate the interrelation between the APOE genotype and a potential neuroprotective effect of galantamine.

## Conclusions

Patients with MCI who were treated with galantamine demonstrated a lower rate of whole brain atrophy, but not of hippocampal atrophy, over a 24-month treatment period, compared with those treated with placebo. This protective effect of galantamine on the whole brain atrophy rate in MCI was only present in APOE ϵ4 carriers.

## Appendix

Independent Ethics Committees or Institutional Review Boards that approved the Gal-Int-11 trial (NCT00236431) per country.

Austria

● Ethikkommission d. Med. Fakultät der Universität Wien, Borschkegasse 8b, A-1090 Wien, Austria

● Ethikkommission der Stadt Wien gem. KAG, AMG und MPG Stabstelle Pharmazie und Medizinökonomie, Schottenring 24, A-1010 Wien, Austria

● Ethikkommission der Med. Fakultät der Universität Innsbruck, Innrain 42, A-6020 Innsbruck, Austria

● Ethikkommission d. Med. Fakultät der Universität Graz, Auenbruggerplatz 29, A-8036 Graz, Austria

Canada

● The University of Western Ontario Research Ethics Office, Dental Sciences Building, London, ON, Canada N6A 5C1

● Comite d’ethique de la recherché Centre Hospitalier Affilie Universitaire de Quebec Hopital de l’Enfant-Jesus Nursing, Cognitive Research Nursing Gerontology Neuropsychology Psychology 1401, 18e Rue, Quebec, QC, Canada G1J 1Z4

● South East Health Care Corporation Research Review Committee, 1365 MacBeath Avenue, Moncton, NB, Canada E1C 6Z8

● University of British Columbia Clinical Research Ethics Board Office of Research Services Room 210, Research Pavilion, 828 W. 10th Avenue Vancouver, BC, Canada V5Z 1L8

● Institutional Review Board, McGill University Faculty of Medicine, McIntyre Medical Bldg, 3655 Sir Willam Osler, Room 637, Montreal, QC, Canada H3G 1Y7

● Service De Sante, Ethics Research Board SCO Health Services, 75 Bruyere, Ottawa, ON, Canada K1N 5C8

● Conjoint Health Research Ethics Board, 3330 Hospital Drive N.W., Calgary, AB, Canada T2N 4N1

● Research Ethics Committee Room 118, Centre for Clinical Research 5790 University Avenue, Halifax, NS, Canada B3H 1V7

● University of Alberta, Health Research Ethics Board 2J2.27 WMC, University of Alberta, Edmonton, AB, Canada T6G 2R7

● Hopital General du Lakeshore General Hospital, 160 Stillview Pointe Claire, QC, Canada H9R 2Y2

● Hopital Maisonneuve-Rosemont, Pavillion Rachel-Tourigny/Ethics Board, 5415 Blvd. de l’Assomption, Montreal, QC, Canada, H1T 2M4

Finland

● Tutkimuseettinen toimikunta KYS Rakennus 2 G (0-krs), P.O. Box 1777, FIN-70211 Kuopio, Finland

France

● CCPPRB de Rennes CHU Pontchaillou, Pavillon Clémenceau, 35033 Rennes, France

Germany

● Medical Ethics Committee II of Faculty for Clinical Medicine, Mannheim Ruprecht-Karls-University Heidelberg, Maybachstraße 14–16, D-68169 Mannheim, Germany

● Ethics Committee of Free University Berlin, University Clinic Benjamin Franklin, Hindenburgdamm 30, D-12200 Berlin, Germany

● Ethics Committee of Albert-Ludwigs University Freiburg, Elsaesser Straße 2m, House 1A, D-79110 Freiburg, Germany

● Ethics Committee of Medical Faculty of Technical University Munich, Ismaninger Straße 22, D-81675 München, Germany

● Ethics Committee of J.W. Goethe University of Frankfurt/Main Theodor-Stern-Kai 7, D-60590 Frankfurt/Main, Germany

● Ethics Committee of University Ulm, Helmholtzstraße 20 (Oberer Eselsberg), D-89081 Ulm, Germany

● Ethics Committee of Medical Faculty of Friedrich-Alexander-University Erlangen-Nueremberg, Universitaetsstraße 40, D-91054 Erlangen, Germany

The Netherlands

● Central: METOPP, Beethovenlaan 332a, 5011 LN Tilburg, the Netherlands

● Local: METC Noord-Holland. Wilhelminalaan 12, 1815 JD Alkmaar, the Netherlands

● Local: METC Noord-Oost Brabant locatie GZG, kamer L1.143, Tolbrugstraat 11, 5211 RW’s-Hertogenbosch, the Netherlands

● Local: METC West-Brabant, Molengracht 21, 4818 CK Breda, the Netherlands

● Local: Subcommissie voor de ethiek van het mensgebonden onderzoek, Hoogbouw 2.15 De Boelelaan 1117, 1081HV Amsterdam, the Netherlands

● Local: MEC azM/UM Attn: Mr. J. Smeets, secretaris P. Debyelaan 25, 6229 HX Maastricht, the Netherlands

Poland

● Komisja Etyczna Centralny Szpital Kliniczny MSWiA, Wołoska 137, 02–507 Warszawa, Poland

● Niezależna Komisja Bioetyczna Do Spraw Badań Naukowych przy Akademmi Medycznej w Gdańsku, M. Skłodowskiej–Curie 3a, 80–210 Gdańsk, Poland

● Komisja Bioetyczna Przy Akademii Medycznej im. Karola Marcinkowskiego w Poznaniu Fredry 10, 61–701 Poznań, Poland

● Uczelniana Komisja Etyki Badań Naukowych Przy Akademii Medycznej wŁodzi Al. Kościuszki 4, 90–419 Lodz, Poland

● Komisja Bioetyczna Śląskiej Akademii Medycznej Poniatowskiego 15, 40–055 Katowice, Poland

Sweden

● Research Ethics Committee, University of Umeå, SE-901 87 Umeå, Sweden

● Research Ethics Committee, University Hospital, SE-581 85 Linköping, Sweden

● Research Ethics Committee, University Hospital, SE-221 85 Lund, Sweden

● Research Ethics Committee Arvid Wallgrens Backe Hus 7, Box 454, SE-405 30 Göteborg, Sweden

● Research Ethics Committee Faculty of Medicine Karolinska Institute, SE-17177 Stockholm, Sweden

United Kingdom

● MREC (central IEC): Administrator MREC for Wales BRO TAF Health Authority, Cathays Park, Cardiff CF10 3NW, UK

● LREC (local IEC): Southampton and South West Hants Joint Local Research Ethics Committee, Trust Management Offices, Southampton General Hospital, Tremona Road Southampton SO16 6YD, UK

● LREC (local IEC): Swindon Research Ethics Committee, Wiltshire Health Authority, Southgate House, Pans Lane, Devizes SN10 5EQ, UK

● LREC (local IEC): National Waiting Time Centre, Beardmore Street, Clydebank G81 4HX, UK

● LREC (local IEC): Health Board Research Ethics Committee, Boswell House, 10 Arthur Street, Ayr KA7 1OJ, UK

● LREC (local IEC): Bradford Local Research Ethics Committee, Bradford Royal Infirmary, Duckworth Lane, Bradford BD9 6RJ, UK

● LREC (local IEC): Local Research Ethics Committee, North Bristol NHS Trust Head Quarters, Clincal Governance Directorate Executive Team Base, Frenchay Hospital, Bristol BS16 1JE, UK

● LREC (local IEC): Southampton and South West Hants Joint Local Research Ethics Committee, Trust Management Offices, Southampton General Hospital, Tremona Road, Southampton SO16 6YD, UK

USA

● Biomed Research Institute of America, 3110 Camino Del Rio, South Suite A215, San Diego, CA 92108, USA

● North Broward Medical Center Institutional Review Board, 201 E. Sample Road, Pompano Beach, FL 33064, USA

● University of Pittsburgh Review Board, Hieber Building, 3500 Fifth Avenue, Pittsburgh, PA 15213, USA

● Biomedical Research Alliance of NY, LLC 225 Community Drive, Suite 100, Great Neck, NY 11021, USA

● WIRB, 3535 Seventh Avenur Southwest, Olympia, WA 98502–5010, USA

● Oregon Health and Science University Institutional Review Board – L106, 3181 SW Sam Jackson Park Road, Portland, OR 97201–3098, USA

● Institutional Review Board for Human Research, Office of Research Integrity Medical University of South Carolina, 165 Cannon Street, Charleston, SC 29425, USA

● St. John Medical Center Institutional Review Board, 1923 S. Utica Avenue, Tulsa, OK 74104, USA

● University of Texas Health Science Center, 7703 Floyd Curl Drive, San Antonio, TX 78229, USA

● Butler Hospital, 345 Blackstone Blvd, Providence, RI 02906, USA

● Office of Research Administration, Rhode Island Hospital, 593 Eddy Street Providence, RI 02906, USA

● The Ohio State University Research Foundation, 1960 Kenny Road, Columbus, OH 43210, USA

● Human Research Office Brigham & Women’s Hospital, 75 Francis Street Boston, MA 02115, USA

● Committee on the Use of Human Subjects Tulane University Health Sciences Center, 1430 Tulane Avenue, New Orleans, LA 70112, USA

● Emory University Human Investigation Committee, 1256 Briarcliff Road, 4th Floor, South Wing, Atlanta, GA 30306, USA

● Human Investigation Committee, New Haven Conneticut Yale University, 55 College Street, New Haven, CT 06510, USA

● University of Texas Southwestern Medical Center at Dallas IRB, 5323 Harry Hines Blvd, Dallas, TX 75390–8843, USA

## Abbreviations

AD: Alzheimer’s disease; ADAS-Cog: Alzheimer’s Disease Assessment Scale – cognitive subscale; APOE: apolipoprotein E; BBSI: brain boundary shift integral; CI: confidence interval; Gal-Int-11: Galantamine-International-11; MCI: mild cognitive impairment; MRI: magnetic resonance imaging.

## Competing interests

HRB and JSN are employed by Johnson and Johnson. The remaining authors declare that they have no competing interests.

## Authors’ contributions

NDP participated in the coordination of the study, performed the statistical analysis, and drafted the manuscript. WAvdF participated in the coordination of the study, and helped to draft the manuscript. DLK performed the statistical analysis. NCF participated in the coordination of the study, performed the MRI measurements, and helped to draft the manuscript. HRB participated in the coordination of the study, and helped to draft the manuscript. JSN participated in the coordination of the study, and helped to draft the manuscript. FB participated in the coordination of the study, performed the MRI measurements, and helped to draft the manuscript. PS participated in the coordination of the study, and helped to draft the manuscript. All authors read and approved the final manuscript.
